# Sketch of the Geology of Tennessee

**Published:** 1857-04

**Authors:** Richard O. Currey

**Affiliations:** Knoxville, Tennessee


*Sketch of the Geology of Tennessee.* By RICHARD O. CURREY, M. D., Knoxville, Tennessee.

COAL REGION OF TENNESSEE.

Passing by the consideration of any other mineral or ore peculiar to East Tennessee, as well as its agricultural facilities for the present, I will next in order enter upon an examination of the Appalachian coal field lying within the limits of Tennessee.

No subject has caused greater solicitude to English capitalists, than the *possibility* that at no very distant day the coal fields of their island home would be exhausted. So rapidly has the consumption of this mineral increased for the last few years, both from the increase of population, and the extended application of steam, that many of the mines have already failed. Some of the most scientific geologists and intelligent miners were commissioned by Government to investigate this subject, and though they differed as to the probable length of time that it would require to completely exhaust the supply, they all agreed that it was an event which must sooner or later ensue. It is true that generations may pass away before it occurs; but, like a wise goverment, there was immediate prohibition put upon the destruction of the waste coal at the mouths of the pits, and a tax levied on its exportation. The result of such a catastrophe is more easily imagined than described. The thousands of busy hands now engaged with her looms, and spindles, and anvils, and rollers, as well as the millions of active capital employed in keeping them in motion, will be driven elsewhere ere such an event happens; and who cannot see, in the departure of these, the fading glory of the English kingdom, and the loss of her power. This is a striking illustration of the value of this single mineral. And while even a prospect of a failure strikes English hearts with terror, may we not anticipate a bright future from the developments of this mineral in this country. From Maine to Texas, there is scarcely a State that does not possess, to some extent, portions of a coal field; while in many it constitutes the principal formation.

In the Massachusetts and Rhode Island coal fields, there are estimated to be about 500 square miles. In the Appalachian, extending from New York, through Pennsylvania into Ohio, through Virginia, Kentucky and Tennessee, and terminating near Tuscaloosa, Alabama, being 800 miles in length, with an average width of 180 miles, and covering an area of more than 100,000 square miles, there are contained, at a low estimate one million of millions tons of bituminous coal. In the Ohio river coal field, we have another immense basin, embracing nearly the whole of Illinois, southern part of Indiana, and extending across the Ohio into Kentucky; the entire area being not less than 55,000 square miles. In Michigan, including about two-thirds of the State, another field has been explored of 12,000 square miles. In Missouri and Iowa, is still another of 50,000; while Arkansas and Texas each contain important fields. So that, supposing those 250,000 square miles of coal deposits to have an average thickness of 50 feet, we have no less than *three and a-half millions of cubic miles* of coal in the Union.

Two classes of coal are found in our country—the bituminous and the *non*-bituminous, or anthracite. The latter class is found only to a limited extent; being the kind obtained in eastern Pennsylvania, and is said to have been found in East Tennessee. Though these two classes possess striking marks of difference, yet they are to be referred to the same origin. The same fossil plants have been found in each; and as anthracite is coal without bitumen, there has been observed a gradual increase of this property towards the western limit of the Appalachian field. The anthracite has been debituminized by its contiguity to the primordial rocks, and to its having been subjected to a great pressure during its formation. It is similar to, and is, in fact, natural coke.

The Tennessee coal field, being part of the Appalachian, is embraced within the limits of the counties of Claiborne, Anderson, Morgan, Scott, Fentress, Campbell, Overton, Grundy, Van Buren, White, Franklin, Bledsoe, Marion, Hamilton, and Rhea, composing the range of Cumberland mountains. These mountains are again subdivided, with local names attached. The eastern base of these mountains, so far as I have ascertained, rest upon the inclined strata of East Tennessee—though in some places there intervenes a table land of the siliceous stratum and old red sandstone. As we cross the mountains, and in passing through the Sequatchee valley, we notice on its eastern side the inclined strata peculiar to East Tennessee; while across the Sequatchee river and on the western side, a hard, white limestone is found, lying horizontally and overlapping the other strata. This peculiarity is observable throughout this rich valley—being traced as far up as Crab Orchard. Here limestone ledges present a striking contrast with the general character of the surrounding country and may be regarded as a wise provision in the midst of these sandstones and sandy soils. This limestone possesses a peculiar structure—being composed of an infinite number of egg-like fossils; hence it has been termed *oolite* limestone; but it possesses another fossil—the *pentremite*—in as great abundance, and it is also called the *pentremital limestone.*

This limestone is also well displayed all along the western declivity of the mountains, intervening between the shales and sandstones of the coal measures, and the old red sandstone of Devonian system below. Its absence on the eastern, and its unfailing presence on the western declivity, is a striking peculiarity. From this *pentremital* limestone to the summit of the mountains are included the coal measures. They consist of strata of coal, shale, sandstone and conglomerates alternating with each other; there being sometimes from four to six of such series in one elevation, but of varying thicknesses. While on the western declivity of these mountains, the coal occupies almost uniformly a horizontal position, on the eastern the entire strata of coal, shale, sandstone, &c., appear to have been disturbed in some places, being tilted up into an inclined position, while in others no such action appears to have taken place, as they retain the horizontal position.

As this is a subject of interest to our citizens, I have made an analysis of several specimens of coal placed in my hands, giving, also, other analyses made at different places by other persons.

The following is the per centum of each specimen:

| LOCALITY. | CARBON. | VOLATILE MATTER. | ASHES. |
|---|---|---|---|
| 1. Pittsburg, | 55 | 38 | 7 |
| 2. Trade Water, Ohio River, | 69,05 | 21 | 9,95 |
| 3. Rock Spring, Ky., | 80,56 | 10 | 9,44 |
| 4. Ohio River, near Caseyville, | 69,58 | 19 | 11,47 |
| 5. Addison's br., Cumberl'd mt., | 83,22 | 9 | 7,78 |
| 6. Anderson Co., E. Tennessee, | 82 | 10 | 7 |
| 7. Crow Creek, | 77,70 | 14 | 8,30 |
| 8. Sewanee Mining Company, | 79,56 | 14,21 | 6,25 |
| 9. Kimbrough's, Roane County, | 71 | 17 | 12 |
| 10. Gillenwater's, Rhea County, | 69 | 14 | 17 |
| 11. Alabama, Tuscaloosa, | 80,96 | 12,96 | 6 |

The analysis of No. 1 was obtained from Professor Johnson's Tables—Nos. 9 and 10 from Professor Troost's Reports, and No. 11 from Professor Tuomey's Report. No. 5 is the variety of coal brought to the Nashville market, and is of a compact structure, and possessing iridescent colors—No. 3 is from a new mine on Trade Water, Ky., 18 miles from the Ohio River.

Though it is known that the range of the Cumberland mountains belongs to the coal measures, yet so little has been done for its actual exploration, that it is impossible to say where, in particular, coal is to be found. So important an element is it to become in our State welfare, that it is highly necessary that a thorough examination of this field should be made, with accurate analysis, accompanied with a special map, and sections of the entire region.

In the table of analyses I have presented specimens from those localities which are most abundantly worked. There are many others opened for the supply of their immediate neighborhood.

In the *Raccoon* mountains, on the line of the Nashville & Chattanooga Railroad, from whence it is obtained for transportation into Georgia, the coal is similar to that produced from the Sewanee mines—being soft and very readily crumbling into powder. It is admirably adapted for manufacturing purposes.

In the *Sequatchee valley* many valuable banks are opened, the coal from which is of a very superior quality. The construction of a branch railroad to the head of this valley would open to market one of the most productive valleys, as well in agriculture as in coal, to be found in the State. In the vicinity of Pikeville alone there are no less than six banks, all of excellent quality.

In *Walden's ridge*, which separates the Sequatchee from the Tennessee, coal seams are found extending from one declivity to the other; that on the eastern declivity affording the coal for the supply of the Chattanooga market.

The coal which is brought from Poplar creek and other localities in Anderson county, for clearness, purity and value, surpasses any coal that we have yet examined. In many instances it is beautifully iridescent. It is this coal which is brought to the Knoxville market, a distance of sixteen miles by wagon, and by water a distance of about two hundred.

In *Cross mountain* and Elk river valley, the same valuable banks are found. To the west in *Morgan county*, there is an inexhaustible supply, only awaiting some facility for transportation to market, to render these excellent stock-raising lands as valuable for their mineral resources.

The opening of these banks, and the construction of railroads for its speedy and constant transmission to points, both East and West, would, in a few years, tell upon the industrial pursuits of our citizens. The construction of such a road as is contemplated in the Nashville and Knoxville Railroad, and especially if continued to the Mississippi river, would draw out from these rich store houses, treasures that would inspirit the loom and the anvil with new life, and accelerate the speed of the plough. The construction also of the Kentucky Railroad, necessarily passing through the rich coal and iron banks of Anderson, promises a bright future for the manufacturing interests of East Tennessee. This is a subject in which the whole State feels a deep interest, relating, as it does, to State aggrandizement and domestic comfort. Placed in juxtaposition with the iron and the copper, no one can fail to see what the results of their development would be. Not only would capital flow into the country, but population also; until the State would become the Keystone of the South, as Pennsylvania is of the North

In these coal measures, good millstones can be obtained from the conglomerates, and grindstones from the sandstones; while the strata of reddish and of white clay would answer admirably for burning into fire brick and fire stones.—*Southern Journal Medical and Physical Sciences.*

